# Three-Dimensional Cone Beam Computed Tomography (CBCT)-Derived Soft Tissue Changes in Patients with Unilateral Cleft Lip, Alveolus, and Palate with Midfacial Deficiency after 1.5 Years of Bone-Anchored Maxillary Protraction

**DOI:** 10.3390/jcm13102890

**Published:** 2024-05-14

**Authors:** Ralph M. Steegman, Annemarlien F. Klein Meulekamp, Anne-Marie Renkema, Krista I. Janssen, Anne Marie Kuijpers-Jagtman, Yijin Ren

**Affiliations:** 1 Department of Orthodontics, University Medical Center Groningen, University of Groningen, Hanzeplein 1, 9713 GZ Groningen, The Netherlands; r.m.steegman@umcg.nl (R.M.S.); a.f.kleinmeulekamp@gmail.com (A.F.K.M.); a.renkema@umcg.nl (A.-M.R.); k.i.janssen@umcg.nl (K.I.J.); y.ren@umcg.nl (Y.R.); 2 Zijlweg Orthodontie, Orthodontic Private Practice, Zijlweg 148B2, 2015 BJ Haarlem, The Netherlands; 3 Department of Orthodontics and Dentofacial Orthopedics, School of Dental Medicine, University of Bern, Freiburgstrasse 7, CH-3010 Bern, Switzerland; 4 Faculty of Dentistry, Universitas Indonesia, Campus Salemba, Jalan Salemba Raya No. 4, Jakarta 10430, Indonesia

**Keywords:** cleft palate, soft tissue, cone beam computed tomography, bone anchor, malocclusion, angle Class III

## Abstract

**Background:** Bone-anchored maxillary protraction (BAMP) aims to correct midfacial deficiencies, with proven positive skeletal changes without potential unwanted side effects. However, the influence of BAMP treatment on facial soft tissues, particularly in subjects with complete unilateral cleft lip, alveolus, and palate (CUCLAP), remains unclear. **Methods:** This single-center longitudinal cohort study examined the effects of 1.5 years of BAMP treatment on facial soft tissues in growing subjects with complete unilateral cleft lip, alveolus, and palate. The sample consisted of 25 patients, age range 9.7 to 12.6 years. Three-dimensional surface models derived from CBCT scans were superimposed on stable structures of the anterior cranial base and on the occipital area posterior of the foramen magnum to assess three-dimensional changes due to growth and BAMP therapy. **Results:** The results revealed a moderate positive correlation (Pearson’s correlation coefficient from 0.203 to 0.560) between changes in hard tissue and soft tissue; some correlations were found to be weak (<0.300). Linear changes in soft tissue following BAMP were in the same direction as skeletal changes, showing downward, forward, and outward displacement. The only exception was in the vertical dimension. The lower facial third showed a slight but significant reduction, mainly in lip length (−1.2 mm), whereas the middle facial third showed a small increase (1.1 mm). **Conclusions:** It was concluded that during BAMP, soft tissue changes occur in the same direction as skeletal changes, although with a larger variability and less pronounced effects.

## 1. Introduction

In growing subjects with an orofacial cleft, craniofacial growth and development are often hindered, predominantly manifesting as maxillary deficiency and skeletal Class III malocclusion. Maxillary growth is affected in all three dimensions—sagittal, transverse, and vertical—impacting both hard and soft tissue development [1,2]. In patients with a Class III malocclusion without a cleft, facemask therapy used to be the primary orthopedic intervention. However, it has been shown in patients with and without a cleft that the treatment’s effect on the maxilla in the long term is minimal, and that it is associated with downward and backward rotation of the mandible [3,4]. Moreover, tooth-anchored maxillary protraction can induce changes in the proclination of the upper and lower incisors [5].

Subsequently, bone-anchored maxillary protraction (BAMP) became popular as a treatment modality for Class III malocclusion. Previous studies compared bone-borne and tooth-borne maxillary protraction in non-cleft subjects with Class III malocclusion, demonstrating that bone-borne protraction achieved more forward maxillary growth with fewer dental changes and less occlusal plane rotation [6]. A recent systematic review compared the efficacy of different bone-borne protraction protocols and concluded that bone-anchored maxillary protraction (BAMP) showed favorable skeletal effects with a larger advancement of point A and less clockwise rotation of the mandible compared to tooth-borne protraction [7,8]. These outcomes are supported by our previous studies and those from other cleft centers on BAMP in subjects with a complete unilateral cleft lip, alveolus, and palate [9,10,11].

In addition to skeletal changes, facial soft tissue alterations are important considerations in treatment planning [12,13,14]. To date, no studies have reported the impact of BAMP on 3D facial soft tissue in patients with clefts based on CBCT scans. Whether the favorable outcomes observed in the zygomatic–maxillary complex and the mandible for the correction of Class III malocclusion and skeletal jaw relationship extend to significant improvement in the facial soft tissue profile remains unanswered.

The aim of this longitudinal cohort study was to assess the effect of BAMP therapy on facial soft tissues in growing children with complete unilateral cleft lip, alveolus, and palate (CUCLAP) and midfacial deficiency with Class III malocclusion. Three-dimensional (3D) surface models derived from cone beam computed tomography (CBCT) scans were employed to analyze soft tissue changes after 1.5 years of BAMP treatment. Additionally, this study explored correlations and ratios between 3D changes in facial soft tissues and corresponding alterations in hard tissues, as well as the predictive values of age, gender, and ethnic background in the treatment outcomes.

## 2. Materials and Methods

### 2.1. Trial Registration and Ethical Approval

This single-center longitudinal cohort study was registered at The Netherlands National Trial Registration (TC 6559). Ethical approval was granted by the Medical Ethics Committee at the University Medical Centre Groningen (Clinical Study Register 201700423, ethical approval METc 2017/318). All patients signed a written informed consent prior to the start of the orthodontic treatment.

### 2.2. Study Subjects

A power analysis on the minimal number of subjects needed with a power of 80% and *p* < 0.05 showed that 21 participants were needed to detect a difference before and 1.5 years after BAMP on A-point soft tissue region of interest (A ROI) from CBCT surface models and to detect a difference on A ROI between hard and soft tissue surface models.

### 2.3. Inclusion Criteria

The inclusion criteria were the same as in our previous study [9,10,15], namely: non-syndromic with a complete unilateral cleft lip, alveolus, and palate; either a sagittal overjet between +2 and −5 mm, an ANB angle < 0° or a Wits < 0 mm, or having both dental and skeletal features; secondary alveolar bone grafting prior to BAMP; no or minor dental alignment in the maxillary arch in preparation for bone grafting prior to BAMP; both lower permanent canines have been erupted. The exclusion criteria were as follows: additional craniofacial anomalies; anterior forced bite or functional shift present.

Patients who participated in the study had started the BAMP treatment between January 2015 and December 2019. All subjects had undergone a series of interdisciplinary treatments at the Cleft Center North in the Netherlands under the same set of protocols. Orthodontic treatment was performed by the same orthodontist (Y.R.) for all subjects at the Department of Orthodontics of the University Medical Center Groningen (UMCG).

### 2.4. Bone-Anchored Maxillary Protraction (BAMP)

Bollard mini plates (Tita-Link, Brussels, Belgium) were placed following a standard protocol at the Department of Oral and Maxillofacial Surgery at the same hospital when patients were approximately 11 years old. The placement of four Bollard bone plates was performed by one experienced operator (maxillofacial surgeon specializing in cleft procedures). Two Bollard bone plates (Tita-Link, Brussels, Belgium) were positioned on the zygomatic buttresses, and two were placed buccally on the anterior part of the mandible between the lateral incisor and canine. All four Bollard bone plates were placed in a single session under general anesthesia [9,16].

Three weeks after placement, maxillary protraction was started with intermaxillary elastics, with an initial force of 150 g on each side and gradually increasing to 200–250 g after 2 to 3 months. Patients were deemed to wear the elastics for 24 h per day, including mealtimes, and to change them at least once a day. A removable bite plate was used for 3–5 months in five patients to relieve occlusal interference. Minor dental alignment was initiated in all subjects approximately 10 months after the initiation of BAMP with a fixed appliance only in the upper arch.

### 2.5. CBCT Acquisition

CBCT scans were taken before the start of BAMP for diagnostic reasons (T0) and approximately 18 months after the start of maxillary protraction (T1). Subjects were positioned in the CBCT scanner in a sitting position with the Frankfurt horizontal (FFH) plane parallel to the floor and centrally positioned in the Field of View (FoV) with the aid of the laser alignment lights. The dentition was in maximal occlusion during acquisition. Subjects were instructed not to move, not to swallow, and to breathe normally during acquisition. CBCT scans were performed using the Planmeca ProMax 3D Mid (Planmeca Oy, Helsinki, Finland), set at 90 kV and 20.25 mAs using a 170 × 200 mm FoV as described in detail in our previous studies [9,10,15]. The ultra-low dose protocol was applied, according to the manufacturer’s guidelines. The effective dose was calculated as 0.04 mSv.

### 2.6. Segmentation, Superimposition, and Measurement of the CBCT 3D Surface Models

**Figure 1:**
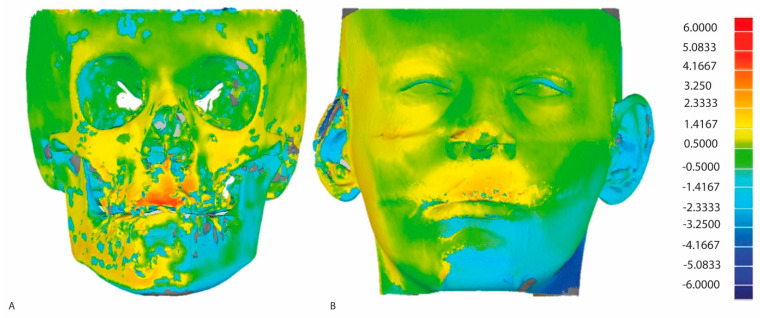
Colormaps of soft and hard tissues in the 3D surface models obtained using CBCT. (**A**) hard tissue colormap; (**B**) soft tissue colormap. The scale bar indicates the direction and mm of the displacement between T0 and T1.

The DICOM data were exported into the specialized software Mimics10.01 (Materialise, Leuven, Belgium, V10.2.1.2) for both hard and soft tissue segmentation based on double thresholds. Two 3D surface models were created, one with only a hard tissue surface model and one with the hard and soft tissue surface models combined. The 3D surface models were imported in Geomagic (version 2013.0.1.1206, Geomagic Solutions, Rock Hill, CA, USA) for 3D superimposition and comparison between T0 and T1. The T0 surface models were used as the reference and the T1 models as test models. Stable hard tissue structures in the anterior cranial fossa and the occipital area posterior of the foramen magnum were selected for superimposition [17]. This superimposition was performed twice. First, the hard tissue models were superimposed, from which a colormap of skeletal changes was made (Figure 1). Measurements were performed on a colormap comprising the two superimposed models at various ROIs: Nasion (Na), left and right zygomatic processes (Zyg), left and right Exocanthion, and A point and B point. The RIOs had an area of 4.5 mm^2^ around every anatomical landmark.

Subsequently, the 3D coordinates (x, y, and z) of these ROIs were used for the second superimposition, which combined the surface models with both the hard and soft tissue models. The same stable structures were used in the second superimposition, from which a second colormap was created for soft tissue.

**Figure 2:**
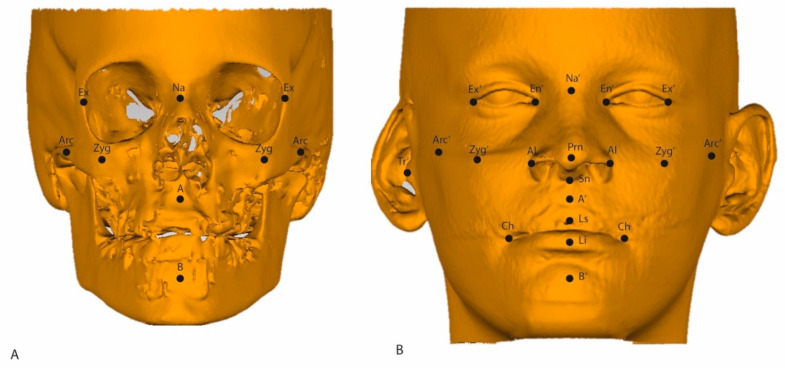
(**A**) Illustration of the ROIs on the 3D hard tissue surface model; (**B**) 3D soft tissue surface model (**B**).

In Table 1, a description of the soft tissue ROIs used in the present study is provided. A description of the hard tissue ROIs has been previously reported [9,10]. In Figure 2, hard tissue and soft tissue ROIs are illustrated on the respective surface models. Every measurement was performed twice by one observer (A.F.K-M.), with at least one week in between measurements.

**Figure 3:**
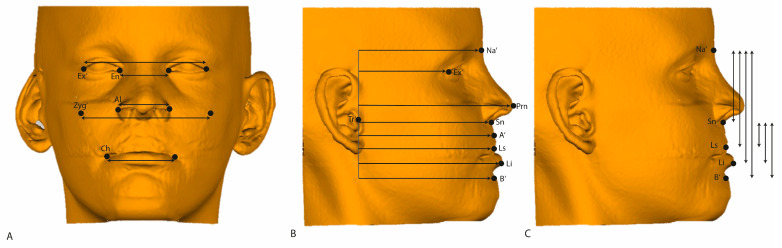
Illustration of linear measurements on a 3D soft tissue surface model. (**A**) Frontal view: horizontal measurements between symmetrical landmarks. (**B**) Lateral view: horizontal measurements from the sagittal landmarks to the line through Tr’ perpendicular to Frankfurt horizontal. (**C**) Lateral view: vertical measurements between the sagittal landmarks. The same landmarks as those illustrated in Figure 2B are used.

Based on the three-dimensional coordinates of the soft tissue ROIs, linear measurements were calculated. In the transversal direction (X), the Exochantion, Endochantion, Alare, Zygoma, and cheilion were used. Transversal changes between the left and right were calculated by extracting both coordinates. In the vertical direction (Z), soft tissue Nasion and Subnasale were used as reference points. In the sagittal direction (Y), changes were calculated based on a reference line, a line passing through the soft tissue tragus perpendicular to the Frankfurt horizontal plane. In Figure 3, all linear changes are illustrated in different views.

### 2.7. Statistics

A power analysis was performed a priori using G*Power 3.1, with an effect size of ρ = 0.5 with a power of 0.8 [18]. Statistical analyses were performed with SPSS for Windows (version 28.0; IBM, Armonk, NY, USA).

To test for the intra-observer reliability of repeated measurements, an intraclass correlation coefficient was calculated, with an ICC between 0.75 and 0.90 indicating good reliability and one between 0.50 and 0.75 indicating moderate reliability.

Means and SDs were calculated for all variables at T0 and T1. Mean increments, SDs, and 95% confidence intervals (CIs) were calculated for T1-T0. Data were tested for normality using the Kolmogorov–Smirnov test. A paired sample T-Test was used to test the increments and for comparison between soft and hard tissue changes. The level of significance all tests was set at *p* < 0.05.

To determine the correlations between hard and soft tissue changes, the Pearson correlation for coefficient was calculated, with a correlation coefficient R value of 0.50–0.70 indicating moderate correlation and one above 0.70 indicating high correlation.

Additionally, both univariate and multivariate regression analyses were conducted to investigate patient-related factors—namely, age at the start of BAMP, gender, and ethnic background—predicting the outcomes of facial soft tissue treatment.

## 3. Results

### 3.1. Sample

Twenty-eight consecutively treated patients were recruited. Three patients were excluded because of CBCT acquisition errors. Finally, a total of 25 patients were included in this study; 17 were male and 8 were female; 19 were Caucasian and 6 non-Caucasian (Chinese). The subjects had a mean age of 11.3 ± 0.4 years at T0 ranging from 9.7 to 12.6 years. The mean age at T1 was 12.8 ± 0.4 with a range of 11.5 to 14.5. Mean treatment duration was 1.5 ± 0.3 years. No patients were lost to follow-up. All subjects exhibited a high level of compliance based on self-reporting.

### 3.2. Intra-Observer Reliability

The ICC for overall soft tissue displacement ranged from 0.762 to 0.945, indicating high reliability. Only the ROI of the left Endocanthion and Left Alar had a lower interclass correlation (0.617 and 0.591). For hard tissue, the ICC ranged from 0.746 to 0.923.

### 3.3. Soft Tissue and Corresponding Hard Tissue Changes

In Table 2, the 3D changes to soft and hard tissues at the corresponding ROIs are presented. An average overall change of 1.60 mm in hard tissue is observed at the A ROI, corresponding to a significantly higher overall change of 2.58 mm in soft tissue at the A ROI (*p* < 0.001). The changes in both soft and hard tissues at the A ROI are attributed to their forward displacement, with negligible changes in the vertical dimension. In the zygomatic region, changes are observed in all three dimensions. Average overall changes of 2.21 mm (left) and 2.19 mm (right) are observed in hard tissue, corresponding to favorable but significantly lower overall changes of 1.72 mm (left) and 1.51 mm (right) at the soft tissue level (*p* = 0.008 and *p* < 0.001). On the zygomatic arch, an outward displacement was found (1.2 ± 0.8 mm), accompanied by a slight forward displacement. Changes in hard and soft tissues showed very low correlations. Only at the A ROI and B ROI were moderate correlations (R = 0.560 and 0.542, respectively) detected between hard and soft tissue changes.

### 3.4. Ratios of Soft Tissue and Corresponding Hard Tissue Changes

**Figure 4:**
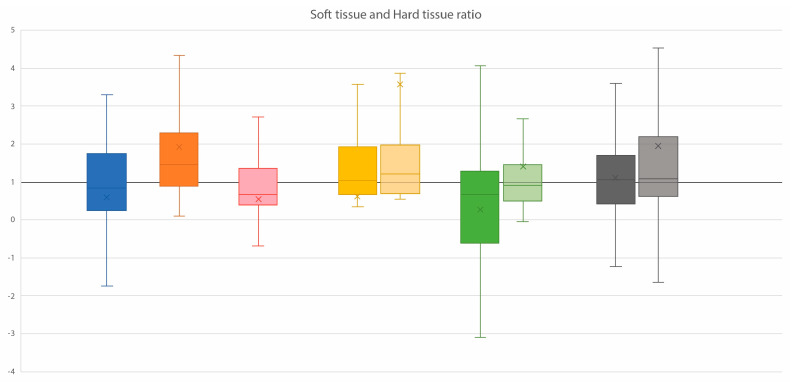
A boxplot of ratios between the soft and hard tissue changes at the respective ROIs. The Y-axis indicates the ratio, and the x on the plot represents the mean value, with the horizontal line in the box indicating the median.

The ratios of soft and hard tissue changes in individual subjects are illustrated in Figure 4. Approximately 75% of the subjects showed a ratio above 1 at the A ROI, indicating that in these subjects, every 1 mm displacement of hard tissue resulted in more than 1 mm displacement of soft tissue. The zygoma regions showed similar ratios on the left and right sides, with about 50% of the ratios being between 0.8 and 2. At the B ROI, more than 50% of the ratios were below 1, indicating that in these subjects, every 1 mm displacement at the B ROI resulted in less than 1 mm displacement at the corresponding soft tissue site.

The nose moved forward and outward, as indicated by the Prn and Al left and right. The lips exhibited more forward than downward movement, as indicated by Ls, Li and Sn’, and Ch left and right. The two inner commissure points of the eye fissure displayed slight forward and outward movement, as indicated by En’ left and right (Table 3).

In Table 4, all linear measurements described in Figure 3 are presented. The increments for the transversal dimensions were all statistically significant, with the mean change ranging from 0.8 to 2.2 mm. The percentual change was the largest for alar width (AlL-AlR), which increased by 6.5% as compared to its width at T0. In the vertical direction, lip length exhibited slight but significant decreases in all relevant dimensions, namely the ROIs Sn’-Ls (Subnasale–Labrale Superius), Sn’-Li (Subnasale–Labrale Inferius), and Sn’-B’ (Subnasale–B). The percentual decrease (9.2%) was the largest for Sn’-Ls. Significant positive increments were observed for all sagittal dimensions, ranging from 1.0 to 3.1 mm, especially in the maxilla, indicating a forward and downward displacement of the face. The largest percentual increases were found for Tr’-Prn (3.1%) and for Tr’-A’ (3.1%).

**Figure 5:**
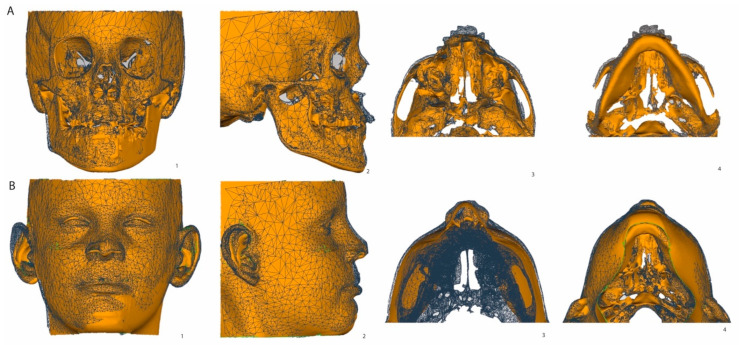
A patient example of corresponding hard and soft tissue changes between T1 (in mesh) and T0 (in solid orange) in different views. (**A**) Superimposition on hard tissue surface model. (**B**) Superimposition on soft tissue surface model. 1: frontal view; 2: lateral view; 3: cranial view; 4. caudal view.

**Figure 6:**
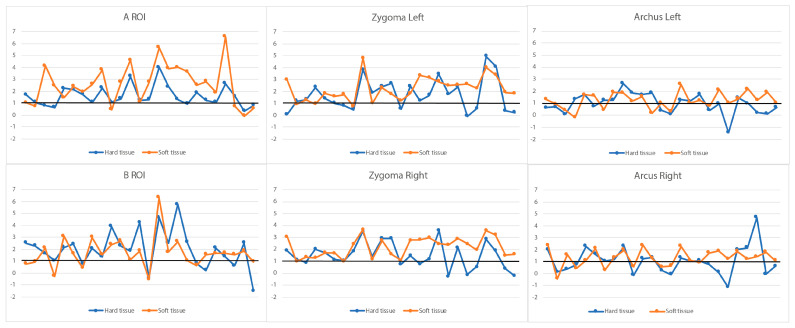
Hard and soft tissue changes in the individual subjects at the respective ROIs between T1 and T0. Orange: soft tissue changes. Blue: hard tissue changes. The Y-axis indicates changes between T1 and T0 for individual subjects in mm, with the individual subjects randomly aligned along the X-axis.

Changes to the corresponding hard tissue and soft tissue between T1 and T0 are illustrated in different views in Figure 5. Generally, the displacement of soft tissue follows hard tissue in the same direction, especially in the zygomatic–maxillary complex. A boxplot of the corresponding ROIs from all individual subjects (Figure 6) shows large variations in all ROIs in both hard tissue and soft tissue.

### 3.5. Regression Analyses of Patient-Related Factors in Treatment Outcomes

Exploratory regression analyses were performed with patient age at T0, gender, and ethnic background as predictors. None of the predictors—whether considered individually or in combinations of more than two variables—were found to have a statistically significant effect on facial soft tissue changes at T1.

## 4. Discussion

Favorable hard tissue changes following 1.5 years of BAMP in growing subjects with unilateral cleft lip, alveolus, and palate with a midfacial deficiency and Class III malocclusion were demonstrated in our previous study and were mainly attributed to a forward and outward displacement of the zygomatic–maxillary complex [9,19]. However, evidence is lacking regarding the three-dimensional effect of BAMP on facial soft tissues. Furthermore, the relationship between the changes in hard and soft tissues remains uncertain. The present study presents the initial findings on the effects of 1.5 years of BAMP on three-dimensional changes in facial soft tissues. The results demonstrate favorable displacements at the ROIs within the midsagittal plane. Moreover, moderate correlations were observed between changes to soft and hard tissues at the A and B ROIs. Gender, age, and ethnic background had no effect on the degree of displacement.

The correction of a midfacial deficiency with a Class III malocclusion using a facemask, orthodontic fixed appliances, or a combined orthodontic–orthognathic approach frequently results in a posterior tilt of the occlusal plane, resulting in clockwise rotation of the mandible and an increased lower facial height. As shown in our previous study, 1.5 years of BAMP did not induce changes in the cant of the occlusal plane and changes to hard tissue in the vertical dimension were minimal, alongside a marginal reduction in the mandibular Gonial angle [6]. The soft tissue superimpositions supported these findings, suggesting a slight decrease in lower facial height following 1.5 years of BAMP. However, quantitative measurements in the lower face should be interpreted with caution due to the use of a chin support during CBCT acquisition.

Prediction of soft tissue response following hard tissue displacement remains a topic of debate. Two-dimensional cephalometric studies on non-cleft subjects showed between 50% and 79% soft tissue response in the maxilla and between 71% and 81% in the mandible after maxillary expansion and protraction headgear treatment [20]. Sade Hoefert et al. reported soft tissue changes subsequent to rapid maxillary expansion (RME) and facemask therapy in subjects with different types of clefts and Class III malocclusion. Similarly, positive changes were observed in the midface, ranging from 0.5 to 2 mm, and at the A ROI (2.9 mm) [21]. However, the study subjects were substantially younger (5.3 years old), the treatment duration was shorter (9 months), and, notably, soft tissue changes were assessed using 3D photos superimposed on soft tissue structures rather than on stable structures at the anterior cranial base [17]. Elnagar et al. assessed 3D soft tissue changes resulting from BAMP and bone-anchored facemask therapy. Both treatment modalities improved the concave facial profile, with a soft tissue response of about 88% in the upper lip, cheeks, and midface [22].

Reports on soft tissue changes after orthognathic surgery are inconsistent [23]. Soft tissue response after bimaxillary surgery involving maxillary advancement and mandibular setback for the correction of Class III malocclusion varies between 30% and 100% in different anatomical areas [24]. Soft tissue response after Le Fort I osteotomy varies between different surgery protocols [25]. Mandibular setback, on the other hand, demonstrated almost 1-to−1 soft tissue changes at the B-point and Pogonion [26]. Prediction of soft tissue response using surgical planning software often lacks accuracy in specific areas of the face and is therefore unreliable. Moreover, it remains undisclosed how these predictions are obtained by the software or what theoretical model they are based upon [27].

Following 1.5 years of BAMP, more soft tissue displacement than that of the underlying hard tissue was observed in the majority of the study subjects at the A ROI; the corresponding changes were moderately correlated. However, the soft tissue changes were small, and the clinical relevance of these findings should be investigated in a larger sample. Furthermore, the moderate correlation between the soft tissue and hard tissue changes at the B ROI needs to be interpreted carefully, as the identification of the B ROI can be influenced by the chin support used during CBCT acquisition.

During BAMP, the zygomatic process moved not only forward and downward but also outward, resulting in a more prominent midface. This can be considered an advantage in addition to the improvement of the facial profile. In comparison, Le Fort I advancement, which takes place below the zygomatic process, is not able to improve a flat midface, a core feature of many cleft patients [9]. It can, therefore, be argued that BAMP may find applications in more severe Class III malocclusion in growing subjects in whom the primary objective is not to avoid a future osteotomy. Rather, it may aim to gain more midface support and to achieve acceptable dentoalveolar function before a potentially less invasive surgical intervention can take place. However, it should be noticed that the largest percentual change in soft tissue was found for the alar base width (AlL-AlR), which increased by 6.5% (2.2 ± 1.8 mm) during BAMP therapy. Such a widening of the alar base is also found after a conventional Le Fort I osteotomy, compromising the esthetic result [25,28].

One limitation of the current study is the absence of an (untreated) control group due to ethical reasons. It is therefore impossible to discern between the treatment effect and natural growth. While the number of subjects is above the minimal power requirement, the sample size remains small. Substantial individual variations may have obscured the identification of certain underlying changes and of the true predictive value of patient-related factors, such as age at the start of treatment, gender, and ethnic background. Also, the favorable results demonstrated in the present study were small and based on a relatively short follow-up period. Maxillofacial growth was not yet complete at the end of the observation period. A longer follow-up is essential to provide insight into the stability of the achieved results and assess potential treatment gain when BAMP is continued beyond the current observation period. Another point for discussion is that the high level of compliance was self-reported. There is indeed no information about the actual wearing time of the elastics, which could have influenced the individual treatment response.

Another limitation of the current study is that soft tissue changes were measured on surface models produced from CBCT scans using ROIs to indicate incremental changes at specific regions of interest. The findings do not offer insights into volumetric soft tissue changes, and the ICC of some ROIs was only moderate. Moreover, previous studies reported significant differences in measuring various facial features such as vermilion height, mouth width, total facial width, mouth symmetry, soft tissue lip thickness, and eye symmetry. CBCT measurements often result in an underestimation of lip thickness compared to those obtained from 3D stereophotography [29]. Future investigations should consider employing 3D stereophotogrammetry for a comprehensive analysis of 3D facial soft tissue changes, emphasize identifying potential predictive factors, and including more subjects in subgroups. It is important to note that the results reported here are based on a single-center, single-operator study, thus limiting generalizability. Multicenter studies are needed to further investigate effectiveness. Additionally, future focus should be put on patient-reported outcomes (PROs) and assess the ultimate maxillary osteotomy rate once patients have reached adulthood.

## 5. Conclusions

Within the limitations of the current study, our preliminary findings show initial evidence that in growing patients with a unilateral cleft lip, alveolus, and palate and Class III malocclusion, soft tissue changes after BAMP therapy follow the skeletal changes observed, although with a larger variability and less pronounced effects. The predictive values of patient-related factors need further study with a larger power and a longer follow-up.

## Figures and Tables

**Table 1:** Description of the soft tissue ROIs as illustrated in Figure 2.

ROI	Abbreviation	Description
Soft tissue Nasion	Na’	Midpoint on the soft tissue contour of the base of the nasal root at the level of the frontonasal suture.
A point	A’	Most posterior midpoint of the philtrum.
B point	B’	Most posterior midpoint on the labiomental soft tissue contour that defines the border between the lower lip and the chin.
Pronasale	Prn	Most anterior midpoint of the nasal tip.
Subnasale	Sn’	Midpoint on the nasolabial soft tissue contour between the columella crest and the upper lip.
Alare	Al	Most lateral point on each alar contour.
Exocanthion left and right	Ex’	Soft tissue point located at the outer commissure of each eye fissure.
Endocanthion left and right	En’	Soft tissue point located at the inner commissure of each eye fissure.
Labrale Superius	Ls	Midpoint of the vermilion line of the upper lip.
Labrale Inferius	Li	Midpoint of the vermilion line of the lower lip.
Cheilion left and right	Ch	Point located at each labial commissure.
Soft tissue Zygoma left and right	Zyg’	Most anterior party of the left/right zygomatic process; the skeletal, vertical, and transversal coordinates count as a reference.
Soft tissue Arcus left and right	Arc’	Most transversal point of the arcus zygomaticus; the skeletal, vertical, and transversal coordinates count as a reference.
Soft tissue Tragus	Tr’	Most outward-pointed eminence of the external right ear.

**Table 2:** Mean values, standard deviations (mm), and correlation coefficients of changes in hard and soft tissues at the corresponding ROIs. Paired difference indicates the mean and 95% CI of the difference between the mean hard tissue increment and mean soft tissue increment between T1 and T0; *p* value indicates the significance of the paired difference. # indicates that the soft and hard tissue changes are moderately correlated with an R value between 0.5 and 0.7.

ROI		Hard Tissue Increment	Soft Tissue Increment	Paired Difference	95% CI	*p* Value	Correlation
Nasion	Overall	0.89 ± 0.62	0.98 ± 1.35	0.09 ± 1.28	−0.27; 0.45	0.629	0.346
	Horizontal	0.85 ± 0.60	0.94 ± 1.29	−0.09 ± 1.21	−0.43; 0.25	0.604	0.360
	Vertical	0.12 ± 0.18	0.07 ± 0.28	0.05 ± 0.31	−0.04; 0.14	0.241	0.160
A	Overall	1.60 ± 0.94	2.58 ± 1.74	0.97 ± 1.46	0.55; 1.39	**<0.001**	0.560 #
	Horizontal	1.50 ± 0.88	2.27 ± 1.60	−0.77 ±1.36	−1.15; −0.38	**<0.001**	0.552 #
	Vertical	−0.14 ±0.42	−0.44 ±1.08	0.31 ± 1.13	−0.01; 0.63	0.059	0.090
B	Overall	1.69 ±1.42	1.99 ± 1.63	0.30 ± 1.47	−0.11; 0.72	0.154	0.542 #
	Horizontal	1.56 ± 1.32	1.83 ± 1.59	−0.26 ± 1.48	−0.86; 0.16	0.216	0.497
	Vertical	0.45 ± 0.47	0.15 ± 0.85	0.30 ± 0.76	0.09; 0.52	**0.007**	0.454
Zygoma L	Overall	2.21 ± 1.05	1.72 ± 1.40	−0.49 ± 1.27	−0.85; −0.13	**0.008**	0.495
	Horizontal	1.78 ± 0.84	1.54 ± 0.13	0.24 ± 1.14	−0.08; 0.57	0.139	0.483
	Vertical	0.80 ± 0.56	−0.38 ± 0.41	1.19 ± 0.79	0.96; 1.41	**<0.001**	−0.294
	Transversal	0.93 ± 0.54	0.60 ± 0.49	−0.33 ± 0.57	−0.49; −0.17	**<0.001**	0.397
Zygoma R	Overall	2.19 ± 0.90	1.51 ± 1.16	−0.68 ± 1.18	−1.01; −0.35	**<0.001**	0.356
	Horizontal	1.84 ± 0.77	1.32 ± 1.03	0.52 ± 1.05	0.22; 0.82	**<0.001**	0.347
	Vertical	0.68 ± 0.49	−0.32 ± 0.36	1.00 ± 0.67	0.81; 1.19	**<0.001**	−0.187
	Transversal	0.81 ± 0.51	0.61 ± 0.47	0.20 ± 0.51	0.05; 0.35	**0.008**	0.447
Arcus L	Overall	1.24 ± 0.75	0.96 ± 0.87	−0.28 ± 1.03	−0.57; 0.01	0.059	0.203
	Horizontal	0.20 ± 0.17	0.16 ± 0.26	0.037 ± 0.27	−0.4; 0.11	0.335	0.291
	Vertical	0.17 ± 0.25	0.10 ± 0.13	0.079 ± 0.27	0.00; 0.15	**0.043**	0.157
	Transversal	1.22 ± 0.67	0.93 ± 0.84	−0.30 ± 0.92	−0.56; −0.04	**0.026**	0.281
Arcus R	Overall	1.30 ± 0.75	1.07 ± 1.20	−0.23 ± 1.20	−0.57; 0.11	0.182	0.310
	Horizontal	0.25 ± 0.19	0.19 ± 0.27	0.06 ± 0.30	−0.03; 0.14	0.207	0.00
	Vertical	0.13 ± 0.22	0.10 ± 020	0.03 ± 0.30	−0.06; 0.11	0.493	0.155
	Transversal	1.20 ± 0.82	1.04 ± 1.16	0.16 ± 1.16	−0.17; 0.49	0.345	0.345
Exo L	Overall	1.14 ± 0.53	1.02 ± 0.93	−0.12 ± 0.86	−0.36; 0.13	0.342	0.428
	Horizontal	0.88 ± 0.46	0.65 ± 0.66	0.23 ± 0.66	0.04; 0.42	**0.017**	0.362
	Vertical	0.15 ± 0.15	0.27 ± 0.55	−0.12 ± 0.51	−0.27; 0.02	0.101	0.371
	Transversal	−0.46± 0.57	0.56 ±0.61	1.03 ±0.95	0.75; 1.30	**<0.001**	0.326
Exo R	Overall	1.07 ± 0.65	0.81 ± 1.36	−0.26 ± 1.29	−0.63; 0.10	0.157	0.360
	Horizontal	0.85 ± 0.53	0.58 ± 0.95	0.27 ± 0.89	0.01; 0.52	**0.039**	0.368
	Vertical	0.12 ± 0.15	0.07 ± 0.42	0.05 ± 0.41	−0.06; 0.17	0.353	0.222
	Transversal	0.46 ± 0.57	0.56 ± 0.90	−0.10 ± 0.85	−0.34; 0.14	0.407	0.399

**Table 3:** Mean values, standard deviations (mm)m and ranges of the increment (T1-T0) of soft tissue ROIs without a hard tissue equivalent.

ROI	Dimension	Mean ± SD	Range	ROI	Mean ± SD	Range
**Soft tissue ROIs midsagittal plane**						
Prn	Overall	2.78 ± 1.63	0.04; 6.93			
	Horizontal	2.68 ± 1.59	0.04; 6.87			
	Vertical	−0.14 ± 0.39	−1.27; 0.81			
Ls	Overall	2.32 ± 1.92	−0.72; 6.96			
	Horizontal	2.21 ± 1.87	−0.71; 6.86			
	Vertical	0.04 ± 0.55	−2.05; 1.73			
Li	Overall	1.67 ±2.20	−4.23; 5.94			
	Horizontal	1.58 ± 2.10	−4.18; 5.81			
	Vertical	−0.22 ± 0.66	−2.83; 1.20			
**Soft tissue ROIs left**				**Soft tissue ROIs right**		
Al L	Overall	0.83 ± 1.98	−5.27; 4.24	Al R	1.83 ± 2.21	−4.09; 6.86
	Horizontal	0.43 ± 0.88	−1.85; −2.47		0.78 ± 0.96	−1.15; 3.89
	Vertical	−0.07 ± 0.60	−2.25; 1.39		0.02 ± 0.72	−1.81; 3.14
	Transversal	0.62 ± 1.70	−3.53; 5.17		1.54 ± 1.95	−3.75; 6.49
En’ L	Overall	1.01 ± 1.09	−0.96; 3.54	En’ R	0.82 ± 0.91	−0.92; 2.39
	Horizontal	0.65 ± 0.72	−1.79; 2.12		0.58 ± 0.67	−0.68; 1.99
	Vertical	0.15 ± 0.50	−1.32; 1.30		−0.16 ± 0.36	−1.19; 0.44
	Transversal	0.53 ± 0.85	−1.60; 3.20		0.46 ± 0.60	−0.74; 2.06
Ch L	Overall	1.56 ± 1.86	−2.68; 7.29	Ch R	1.99 ± 1.61	−1.35; 6.45
	Horizontal	1.13 ± 1.37	−2.53; 4.60		1.60 ± 1.31	−1.04; 5.80
	Vertical	0.00 ± 1.34	−2.58; 5.47		0.31 ± 1.13	−2.18; 3.13
	Transversal	0.52 ± 0.82	−1.00; 2.52		0.25 ± 0.94	−1.67; 3.57
Arc’ L	Overall	1.24 ± 0.75	−2.00; 3.05	Arc’ R	1.30 ± 0.54	−1.70; 4.76
	Horizontal	0.20 ± 1.7	−0.23; 1.13		0.25 ± 0.19	−0.41; 1.01
	Vertical	0.17 ± 0.25	−0.16; 0.42		0.13 ± 0.22	−0.29; 0.85
	Transversal	1.22 ± 0.67	−1.98; 2.82		1.20 ± 0.82	−1.64; 4.58
Ex’ L	Overall	1.14 ± 0.53	−1.06; 3.87	Ex’ R	1.07 ± 0.65	−1.78; 5.08
	Horizontal	0.88 ± 0.46	−0.76; 2.47		0.85 ± 0.53	−1.70; 3.28
	Vertical	0.15 ± 0.15	−0.94; 1.39		0.12 ± 0.15	−0.94; −0.98
	Transversal	0.49 ± 0.56	−0.62; 2.84		0.46 ± 0.57	−0.87; 3.84

**Table 4:** Pre- and post-treatment linear dimensions and T1-T0 increments (in mm) measured on CBCT-derived soft tissue surface models in the transversal, vertical, and sagittal directions.

Variable	T0	T1	T1-T0 Increment		
	Mean ± SD	Mean ± SD	Mean ± SD	95% CI	*p* Value
**Transversal dimension (mm)**					
Ex’L-Ex’R	94.0 ± 6.7	95.2 ± 6.4	1.2 ±1.2	−1.50; −0.84	<0.001
En’L-En’R	34.0 ± 5.2	35.0 ± 5.0	1.0 ±1.2	−1.33; −0.63	<0.001
AlL-AlR	33.9 ± 3.2	36.1 ± 3.6	2.2 ± 1.8	−2.65; −1.65	<0.001
Zyg’L-Zyg’R	73.3 ± 8.8	74.5 ± 8.8	1.2 ± 0.8	1.41; −0.93	<0.001
ChL-ChR	44.5 ± 5.1	45.2 ± 5.4	0.8 ± 1.3	−1.16; −0.41	<0.001
**Vertical dimension (mm)**					
Na’-Sn’	50.7 ± 3.6	51.9 ± 3.6	1.1 ± 1.3	−1.51; −0.78	<0.001
Na’-Ls	63.9 ± 4.2	63.8 ± 4.3	0.0 ± 0.5	−0.14; 0.16	0.915
Na’-Li	74.9 ± 4.8	74.6 ± 4.8	−0.3 ± 0.7	0.11; 0.50	0.003
Na’-B’	87.5 ± 5.5	87.6 ± 5.6	0.1 ± 0.9	−0.32; 0.19	0.622
Sn’-Ls	13.1 ± 2.5	12.0 ± 3.1	−1.2 ± 1.2	0.80; 1.51	<0.001
Sn’-Li	24.2 ± 3.3	22.7 ± 3.6	−1.4 ± 1.3	1.09; 1.82	<0.001
Sn’-B’	36.8 ± 5.2	35.7 ± 5.4	−1.1 ± 1.4	0.67; 1.50	<0.001
**Sagittal dimension (mm)**					
Tr’-Na’	82.6 ± 6.3	83.9 ± 6.6	1.4 ± 1.5	−3.92; −0.533	0.011
Tr’-Ex’	61.9 ± 4.9	62.9 ± 4.9	1.0 ± 1.2	−1.39; −0.69	<0.001
Tr’-Prn	98.9 ± 6.7	102.0 ± 7.4	3.1 ± 1.7	−3.63; −2.66	<0.001
Tr’-Sn’	88.0 ± 6.5	89.7 ± 6.7	1.6 ± 1.5	−2.04; −1.21	<0.001
Tr’-A’	88.2 ± 8.1	90.9 ± 8.3	2.7 ± 1.7	−3.20; −2.21	<0.001
Tr’-Ls	89.6 ±6.0	92.3 ±6.1	2.7 ± 1.9	−3.08; −2.04	<0.001
Tr’-Li	91.8 ±5.9	93.9 ± 5.8	2.0 ± 2.0	−2.72; −1.56	<0.001
Tr’-B’	86.7 ± 6.7	88.9 ± 6.9	2.3 ± 1.6	−2.80; −1.85	<0.001

## Data Availability

Data unavailable due to privacy or ethical restrictions.

## References

[1] Chen Z.Q., Qian Y.F., Wang G.M., Shen G. (2009). Sagittal Maxillary Growth in Patients with Unoperated Isolated Cleft Palate. Cleft Palate Craniofac. J..

[2] Zheng Z.W., Fang Y.M., Lin C.X. (2016). Isolated Influences of Surgery Repair on Maxillofacial Growth in Complete Unilateral Cleft Lip and Palate. J. Oral. Maxillofac. Surg..

[3] Tindlund R.S., Holmefjord A., Eriksson J.-C.H., Johnson G.E., Vindenes H. (2009). Interdisciplinary Evaluation of Consecutive Patients With Unilateral Cleft Lip and Palate at Age 6, 15, and 25 Years: A Concurrent Standardized Procedure and Documentation by Plastic Surgeon; Speech and Language Pathologist; Ear, Nose, and Throat Specialist; and Orthodontist. J. Craniofac. Surg..

[4] Susami T., Okayasu M., Inokuchi T., Ohkubo K., Uchino N., Uwatoko K., Takahashi-Ichikawa N., Nagahama K., Takato T. (2014). Maxillary Protraction in Patients with Cleft Lip and Palate in Mixed Dentition: Cephalometric Evaluation after Completion of Growth. Cleft Palate-Craniofac. J..

[5] Tome W., Yashiro K., Kogo M., Yamashiro T. (2016). Cephalometric Outcomes of Maxillary Expansion and Protraction in Patients with Unilateral Cleft Lip and Palate after Two Types of Palatoplasty. Cleft Palate-Craniofac. J..

[6] Liang S., Wang F., Chang Q., Bai Y. (2021). Three-Dimensional Comparative Evaluation of Customized Bone-Anchored vs Tooth-Borne Maxillary Protraction in Patients with Skeletal Class III Malocclusion. Am. J. Orthod. Dentofac. Orthop..

[7] Toffol L.D., Pavoni C., Baccetti T., Franchi L., Cozza P. (2008). Orthopedic Treatment Outcomes in Class III Malocclusion. Angle Orthod..

[8] Hu S., An K., Peng Y. (2022). Comparative Efficacy of the Bone-anchored Maxillary Protraction Protocols for Orthopaedic Treatment in Skeletal Class III Malocclusion: A Bayesian Network Meta-analysis. Orthod. Craniofac. Res..

[9] Ren Y., Steegman R., Dieters A., Jansma J., Stamatakis H. (2019). Bone-Anchored Maxillary Protraction in Patients with Unilateral Complete Cleft Lip and Palate and Class III Malocclusion. Clin. Oral. Investig..

[10] Steegman R.M., Meulekamp A.F.K., Dieters A., Jansma J., van der Meer W.J., Ren Y. (2021). Skeletal Changes in Growing Cleft Patients with Class III Malocclusion Treated with Bone Anchored Maxillary Protraction—A 3.5-Year Follow-Up. JCM.

[11] Yatabe M., Garib D.G., Faco R.A.S., de Clerck H., Janson G., Nguyen T., Cevidanes L.H.S., Ruellas A.C. (2017). Bone-Anchored Maxillary Protraction Therapy in Patients with Unilateral Complete Cleft Lip and Palate: 3-Dimensional Assessment of Maxillary Effects. Am. J. Orthod. Dentofac. Orthop..

[12] Antoun J.S., Fowler P.V., Jack H.C., Farella M. (2015). Oral Health–Related Quality of Life Changes in Standard, Cleft, and Surgery Patients after Orthodontic Treatment. Am. J. Orthod. Dentofac. Orthop..

[13] Nolte F.M., Bos A., Prahl C. (2019). Quality of Life Among Dutch Children With a Cleft Lip and/or Cleft Palate: A Follow-Up Study. Cleft Palate-Craniofac. J..

[14] De Queiroz Herkrath A.P.C., Herkrath F.J., Rebelo M.A.B., Vettore M.V. (2015). Measurement of Health-Related and Oral Health–Related Quality of Life among Individuals with Nonsyndromic Orofacial Clefts: A Systematic Review and Meta-Analysis. Cleft Palate-Craniofac. J..

[15] Steegman R., Schoeman A., Dieters A., Jongsma B., Jansma J., van der Meer J., Ren Y. (2022). Three-Dimensional Volumetric Changes in the Airway of Growing Unilateral Complete Cleft Lip and Palate Patients after Bone-Anchored Maxillary Protraction. Am. J. Orthod. Dentofac. Orthop..

[16] Vermeulen F.M.J. (2014). Interceptieve Behandeling van Een Maxillaire Hypoplasie Met Behulp van Botankers. Een Literatuuronderzoek. NTvT.

[17] Gkantidis N., Schauseil M., Pazera P., Zorkun B., Katsaros C., Ludwig B. (2015). Evaluation of 3-Dimensional Superimposition Techniques on Various Skeletal Structures of the Head Using Surface Models. PLoS ONE.

[18] Faul F., Erdfelder E., Lang A.-G., Buchner A. (2007). G*Power 3: A Flexible Statistical Power Analysis Program for the Social, Behavioral, and Biomedical Sciences. Behav. Res. Methods.

[19] De Clerck H., Cevidanes L., Baccetti T. (2010). Dentofacial Effects of Bone-Anchored Maxillary Protraction: A Controlled2 Study of Consecutively Treated Class III Patients. Am. J. Orthod. Dentofac. Orthop..

[20] Ngan P., Hägg U., Yiu C., Merwin D., Wei S.H.Y. (1996). Soft Tissue and Dentoskeletal Profile Changes Associated with Maxillary Expansion and Protraction Headgear Treatment. Am. J. Orthod. Dentofac. Orthop..

[21] Sade Hoefert C., Bacher M., Herberts T., Krimmel M., Reinert S., Göz G. (2010). 3D Soft Tissue Changes in Facial Morphology in Patients with Cleft Lip and Palate and Class III Mal Occlusion under Therapy with Rapid Maxillary Expansion and Delaire Facemask. J. Orofac. Orthop..

[22] Elnagar M.H., Elshourbagy E., Ghobashy S., Khedr M., Kusnoto B., Evans C.A. (2017). Three-Dimensional Assessment of Soft Tissue Changes Associated with Bone-Anchored Maxillary Protraction Protocols. Am. J. Orthod. Dentofac. Orthop..

[23] Möhlhenrich S.C., Kötter F., Peters F., Kniha K., Chhatwani S., Danesh G., Hölzle F., Modabber A. (2021). Effects of Different Surgical Techniques and Displacement Distances on the Soft Tissue Profile via Orthodontic-Orthognathic Treatment of Class II and Class III Malocclusions. Head. Face Med..

[24] Soncul M., Bamber M.A. (2004). Evaluation of Facial Soft Tissue Changes with Optical Surface Scan after Surgical Correction of Class III Deformities. J. Oral. Maxillofac. Surg..

[25] Gil A.P.S., Machado-Fernández A., Guijarro-Martínez R., Hernández-Alfaro F., Haas Jr O.L., De Oliveira R.B. (2022). Le Fort I Osteotomy and Soft Tissue Response: A Retrospective Cohort Study Comparing Three Different Techniques. J. Cranio-Maxillofac. Surg..

[26] Rupperti S., Winterhalder P., Krennmair S., Holberg S., Holberg C., Mast G., Rudzki I. (2022). Changes in the Facial Soft Tissue Profile after Maxillary Orthognathic Surgery. J. Orofac. Orthop..

[27] Olivetti E.C., Nicotera S., Marcolin F., Vezzetti E., Sotong J.P.A., Zavattero E., Ramieri G. (2019). 3D Soft-Tissue Prediction Methodologies for Orthognathic Surgery—A Literature Review. Appl. Sci..

[28] Vasudavan S., Jayaratne Y.S.N., Padwa B.L. (2012). Nasolabial Soft Tissue Changes After Le Fort I Advancement. J. Oral. Maxillofac. Surg..

[29] Metzger T.E., Kula K.S., Eckert G.J., Ghoneima A.A. (2013). Orthodontic soft-tissue parameters: A comparison of cone-beam computed tomography and the 3dMD imaging system. Am. J. Orthod. Dentofac. Orthop..

